# Hop derived flavonoid xanthohumol inhibits endothelial cell functions *via* AMPK activation

**DOI:** 10.18632/oncotarget.10990

**Published:** 2016-08-01

**Authors:** Cristina Gallo, Katiuscia Dallaglio, Barbara Bassani, Teresa Rossi, Armando Rossello, Douglas M. Noonan, Gabriele D'Uva, Antonino Bruno, Adriana Albini

**Affiliations:** ^1^ IRCCS “Istituto in Tecnologie Avanzate e Modelli Assistenziali in Oncologia” Arcispedale S. Maria Nuova, Reggio Emilia, Italy; ^2^ Scientific and Technology Pole, IRCCS MultiMedica, Milan, Italy; ^3^ Department of Pharmacy, Pisa University, Pisa, Italy; ^4^ Department of Biotechnologies and Life Sciencies, University of Insubria, Varese, Italy

**Keywords:** angiogenesis, xanthohumol, AMPK, endothelial cells, polyphenols

## Abstract

Angiogenesis, a process characterized by the formation of new blood vessels from pre-existing ones, is a crucial step in tumor growth and dissemination. Recently, increased attention has been addressed to the ability of flavonoids to prevent cancer by suppressing angiogenesis, strategy that we named “angioprevention”. Several natural compounds exert their anti-tumor properties by activating 5′ adenosine monophosphate-activated protein kinase (AMPK), a key regulator of metabolism in cancer cells. Drugs with angiopreventive activities, in particular metformin, regulate AMPK in endothelial cells. Here we investigated the involvement of AMPK in the anti-angiogenic effects of xanthohumol (XN), the major prenylated flavonoid of the hop plant, and mechanisms of action. The anti-angiogenic activity of XN was more potent than epigallocatechin-3-gallate (EGCG). Treatment of endothelial cells with XN led to increased AMPK phosphorylation and activity. Functional studies using biochemical approaches confirmed that AMPK mediates XN anti-angiogenic activity. AMPK activation by XN was mediated by CAMMKβ, but not LKB1. Analysis of the downstream mechanisms showed that XN-induced AMPK activation reduced nitric oxide (NO) levels in endothelial cells by decreasing eNOS phosphorylation. Finally, AKT pathway was inactivated by XN as part of its anti-angiogenic activity, but independently from AMPK, suggesting that these two signaling pathways proceed autonomously. Our study dissects the molecular mechanism by which XN exerts its potent anti-angiogenic activity, pointing out AMPK as a crucial signal transducer.

## INTRODUCTION

Angiogenesis is the process of formation of new vessels from the pre-existing vasculature during development and wound healing. Pathological angiogenesis is a hallmark of cancer [1], as a crucial process in tumor growth and metastatic dissemination [2, 3]. Strategies aimed at blocking or delaying tumor angiogenesis represent promising therapeutic approaches for cancer prevention and therapy [4, 5]. This concept exemplifies an indirect anti-tumor strategy, aimed at targeting host components rather than tumor cells.

Several natural products received increasing attention as agents for cancer prevention and therapy [6–12] for their anti-proliferative, pro-apoptotic or anti-oxidant activities in tumor cells. These agents also have substantial anti-angiogenic activity [13], giving rise to the concept of “angioprevention” [14, 15]. Xanthohumol (XN), the most abundant flavonoid of the hop plant (Humulus lupulus L.) used to preserve and flavor beer, has gained attention in recent years for its anti-angiogenic and anti-inflammatory activity both *in vitro* and *in vivo* [16–22]. The anti-angiogenic activity has been reported to be mediated by a reduction in the secretion of vascular endothelial growth factor (VEGF) by cancer cells and in the inactivation of AKT/NF-κB pathway in endothelial cells [17–19]. Several natural compounds with chemo-preventive activity, especially flavonoids (such as epigallocatechin-3-gallate, quercetin and resveratrol), exert their anti-angiogenic effects through the activation of AMPK (5′ adenosine monophosphate-activated protein kinase) [23–26] (Table 1). AMPK is involved in many cellular processes, including metabolism, homeostasis regulation, growth, proliferation, apoptosis and autophagy [27–29]. It is a heterotrimeric serine/threonine kinase with a catalytic α-subunit and regulatory β- and γ-subunits and is activated by phosphorylation by multiple kinases [30]. LKB1 (Liver Kinase B1) is the major AMPK kinase under energy-stress conditions leading to an increase in the intra-cellular AMP/ATP ratio [31–34]. However, AMPK can be also activated by other protein kinases, including CaMKKβ (Calcium/calmodulin dependent protein kinase kinase β), which is able to induce AMPK activation following stimuli leading to increased intracellular Ca^2+^ levels [32, 35]. The kinase TAK1 (transforming growth factor β-activated kinase) activates AMPK in response to VEGF and cytokines [28]. In endothelial cells AMPK mediates the response to hormones, vascular mediators, the anti-inflammatory molecule salicylic acid and the anti-diabetic drug metformin [8, 36]. It is able to activate several signaling pathways in order to protect from hypoxia, shear and oxidative stress [37]. The activity of AMPK on the endothelium is exerted through an activating phosphorylation of endothelial nitric oxide synthase (eNOS) at Ser1177 with the subsequent formation of NO (nitric oxide), a central signaling molecule in the regulation of vascular homeostasis [38]. Endothelium-derived NO stimulates blood flow, vascular remodeling and angiogenesis [39].

**Table 1:** Flavonoids and AMPK in endothelial cells

Flavonoids	Source	AMPK activation	Related pathways	References
Epigallocatechin-3-gallate (EGCG)	Green tea, apples, plums, onions, hazelnuts, pecans, cacao	yes	AKT, FOXO1	[24]
Quercetin	Apples, citrus fruits, leafy vegetables, red onions, Dill weed, Capers	yes	IL-1β	[25]
Resveratrol (RSV)	Red wine, grapes, cranberries, blueberries, peanuts	yes	SIRT1	[26]
Xanthohumol (XN)	Beer, hops	?	AKT	[19]

Here, our comparative experiments uncovered higher anti-angiogenic activity of XN compared to the widely investigated epigallocatechin-3-gallate (ECGC). We analyzed AMPK as a possible mediator of XN anti-angiogenic activity. There are so far no reports on AMPK regulation in an anti-angiogenic setting by XN. We show that AMPK is a central mediator of XN anti-angiogenic effects in endothelial cells. We also unveiled molecular details on upstream and downstream players, involving CaMKKβ and eNOS, respectively, and determined that activation of AMPK and inhibition of AKT by XN are independent pathways.

## RESULTS

### Comparison of xanthohumol (XN) and epicallocatechin-3-gallate (EGCG) anti-angiogenic activities

**Figure 1:**
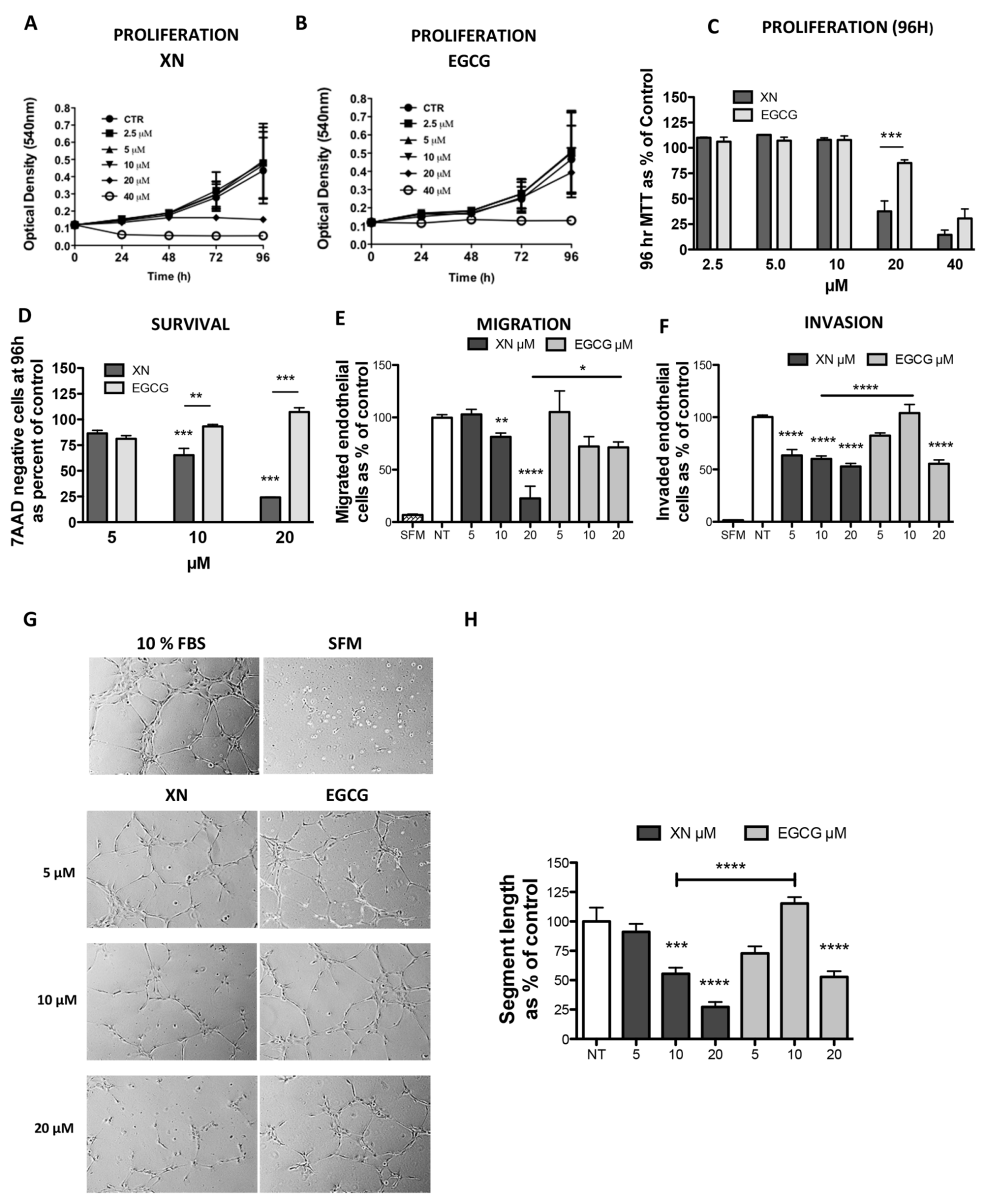
Comparison of anti-angiogenic effects of XN and EGCG **A–C.** HUVEC were treated with various concentrations of XN **(A)** or EGCG **(B)** (0, 2. 5, 5, 10, 20 and 40 μM) **(C)** up to 96h and cell proliferation was determined using the MTT assay. Data are expressed in terms of optical density at 540 nm. **D.** Apoptosis was measured on cells treated with 5, 10, 20 μM XN or 5, 10, 20 μM EGCG at 96 hours by 7AAD staining. 7ADD-negative (viable) cells were determined by FACS analyses and data reported as percent of untreated control. **E, F.** HUVEC cells were pre-treated for 24 hours with increasing concentrations (5, 10 and 20 μM) of XN or EGCG, then seeded in serum free medium in the upper compartment of Boyden chamber. Cell migration (N=5) was measured at 6h and invasion (N=10) at 24h. **G-H** HUVEC were seeded on matrigel pre-coated plates and incubated for 6 hours in complete growth medium to monitor morphogenesis. Microphotographs **(G)** were taken at 10X magnification (N ranging from 3 to 7) and capillary-like tube length was quantified **(H)** by the Angiogenesis analyzer ImageJ tool kit. All experiments were performed three times in duplicate. Data in H are expressed as the mean±SEM of the percentage of control values from independent experiments, with respect to control or as indicated by the bars (****p<0.0001; ***p<0.001; **p<0.01; *p<0.05; One-Way ANOVA).

XN has been shown to exert anti-angiogenic, anti-oxidant and anti-inflammatory activities both *in vitro* and *in vivo* [16–20], suggesting that it could be an angiopreventive compound. Here we evaluated the anti-angiogenic activity of XN compared to the widely recognized anti-angiogenic flavonoid from green tea, EGCG, by analyzing human endothelial cells proliferation, viability and cell functions. MTT assay revealed that XN inhibits cell proliferation/viability more efficiently than EGCG (Figure 1A-1C). Similarly, cell cycle assessment demonstrated that 24h and 48h treatment with XN resulted in a decreased percentage of cells undergoing S-phase and increased cells in the G0/G1 phase, while ECGC displayed no effects. (Supplementary Figure S1A-S1B)

Administration of XN induced apoptosis at high doses (20 μM at 24h) or after a prolonged times of treatment (10 μM at 96h), whereas EGCG did not show effects at the concentrations or times of treatment used (Figure 1D; Supplementary Figure S1C-S1E). We then evaluated the ability of XN and ECGC to interfere with key angiogenic functions, such as migration, invasion and the ability to form capillary-like networks on matrix basement membrane (matrigel). Cells were pre-treated for 24h with XN or EGCG at doses ranging from 5 to 20 μM and migration, invasion and morphogenesis assays were performed. XN significantly reduced endothelial cell migration (Figure 1E) and invasion (Figure 1F). Once again, XN compared to EGCG, was more effective in inhibiting endothelial cell migration at (20 μM concentration) and migration (10 μM concentration). The formation of the endothelial cell networks induced by 10% FBS was inhibited to a greater extent by XN as compared with EGCG (Figure 1G–1H). All our analysis therefore pointed out more powerful anti-angiogenic related activities of XN than EGCG.

### Xanthohumol activates AMPK in endothelial cells

**Figure 2:**
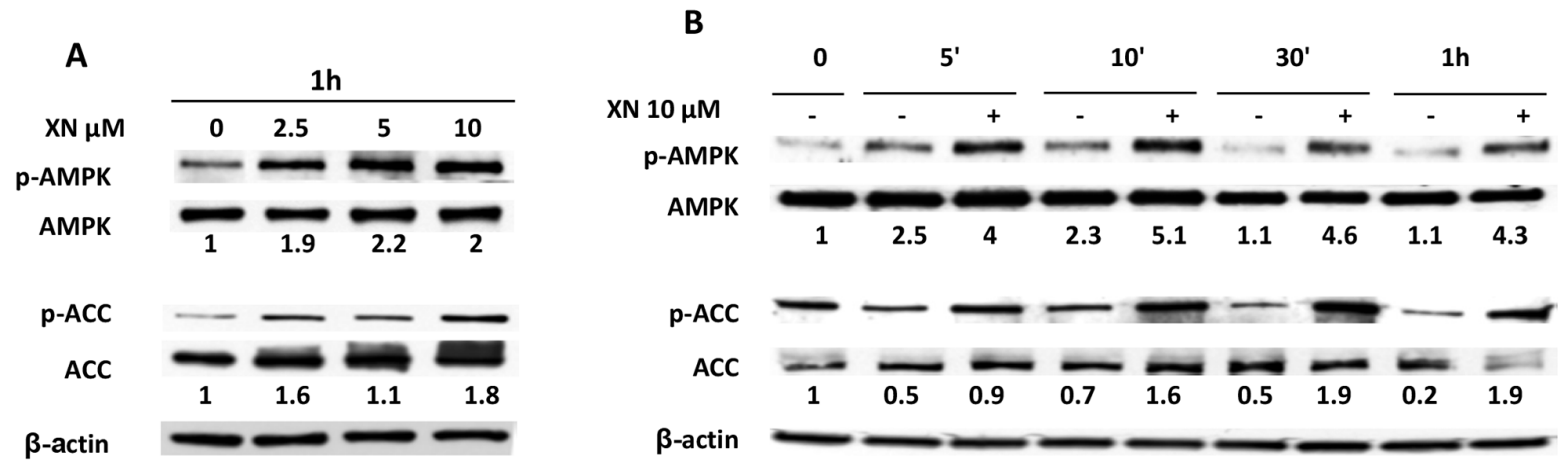
AMPK activation by XN in human endothelial cells **A–B.** HUVEC were treated with increasing doses of XN (2.5-10 μM) for 1 hour **(A)** or treated with 10μM XN at the indicated time points and lysed **(B)**. AMPK activation was evaluated by determination of phospho-AMPK and phospho-ACC levels. AMPK, phospho-AMPK (Thr-172), ACC and phospho-ACC (Ser79) in all lysates were analyzed by western blot. Anti-β-actin antibody was used as loading control.

We previously demonstrated that the anti-angiogenic activity of XN is exerted, at least in part, through the activation of NF-κB and AKT pathways [18, 19]. The involvement of AMPK in XN-induced inhibition of angiogenesis has never being investigated in endothelial cells. We decided to focus on XN at 10 μM or lower concentrations as it reduced endothelial cell functions without affecting cell viability or proliferation (see Figure 1 and Supplementary Figure S1). In order to investigate whether XN is able to activate AMPK in endothelial cells, HUVECs were treated with increasing concentrations of XN for 1h (Figure 2A) or with 10 μM XN in a time ranging from 5 minutes to 1 hours (Figure 2B). AMPK activation was measured by analyzing the level of phospho-AMPK (Thr172) by western blot analysis. XN induced phosphorylation of AMPK in a dose-dependent manner, from 2.5 μM up to 10 μM (Figure 2A). AMPK activation upon treatment with 10 μM XN was observed already at 5 minutes and increased up to 1h (Figure 2B). To elucidate whether XN-induced AMPK activation is of functional relevance, we also analyzed ACC (Acetyl-CoA Carboxylase) phosphorylation at Ser79, a marker of AMPK activity. In line with our hypothesis, XN rapidly induced ACC phosphorylation in a dose-dependent manner, consistently with AMPK activation kinetic (Figure 2A-2B).

### Xanthohumol anti-angiogenic effects are mediated by AMPK

**Figure 3:**
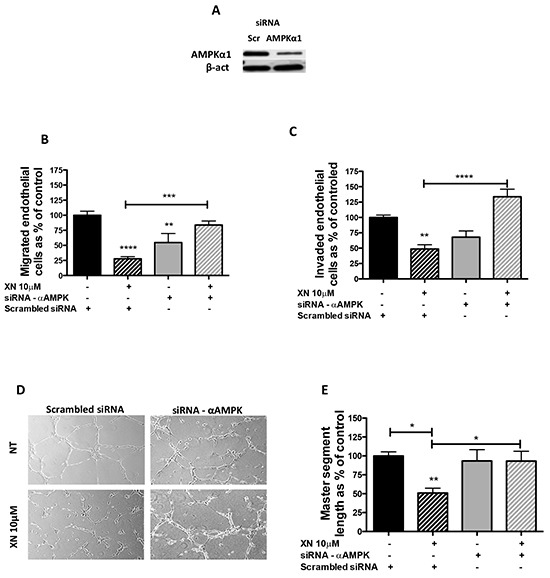
AMPK requirement for XN-induced anti-angiogenic effects **A.** AMPKα1-specific siRNA (siRNA-α AMPK) was used to down-regulate AMPK in HUVEC. Scramble siRNA (scr) treated cells were used as controls. Silencing was evaluated by western blot analysis at 48 hours. **B-E.** 24 hours after scr or siRNA AMPKα1 transfection, cells were treated with 10μM XN for 24h. Viable cells were used to perform migration and invasion assays. AMPKα1 downregulation restored XN-reduced HUVEC ability to migrate **(B)**, invade **(C)** and organize in capillary-like structures **(D)**. Microphotographs were taken at 10X magnification and representative endothelial tubes are shown. Capillary-like tube length was quantified by the Angiogenesis analyzer ImageJ toolkit **(E).** Data are expressed as mean±SEM of the percentage of control values from independent experiments. Statistical significance (****p<0.0001; ***p<0.001; **p<0.01; *p<0.05; One-Way ANOVA) are with respect to control or as indicated by the bars.

We tested whether AMPK activation mediates the anti-angiogenic effects exerted by XN on endothelial cells. AMPK-α1 is the most abundant AMPK isoform expressed by endothelial cells [40]. We therefore down-regulated AMPK-α1 levels by short interfering RNA (siAMPKα1) in HUVEC. Silencing efficiency (70%) was verified by western blot analysis (Figure 3A). Following 24h of transfection with siAMPKα1 or scramble siRNAs, HUVEC were treated with increasing concentrations of XN for 24h. Cell migration, invasion and the ability to form capillary-like networks were then assessed (Figure 3B–3E). We observed that silencing of AMPKα1 reduced endothelial cell migration and invasion (Figure 3B–3C). Further, in line with our hypothesis, silencing of AMPK restored the migratory ability of XN-treated endothelial cells (Figure 3B). Following AMPK silencing, XN was unable to block and even promoted invasion in a not statistically significant manner (Figure 3C). Overall these data suggest that XN-induced inhibition of angiogenesis requires AMPK.

### Xanthohumol effects on AMPK upstream signals (LKB1 and CaMKK β)

**Figure 4:**
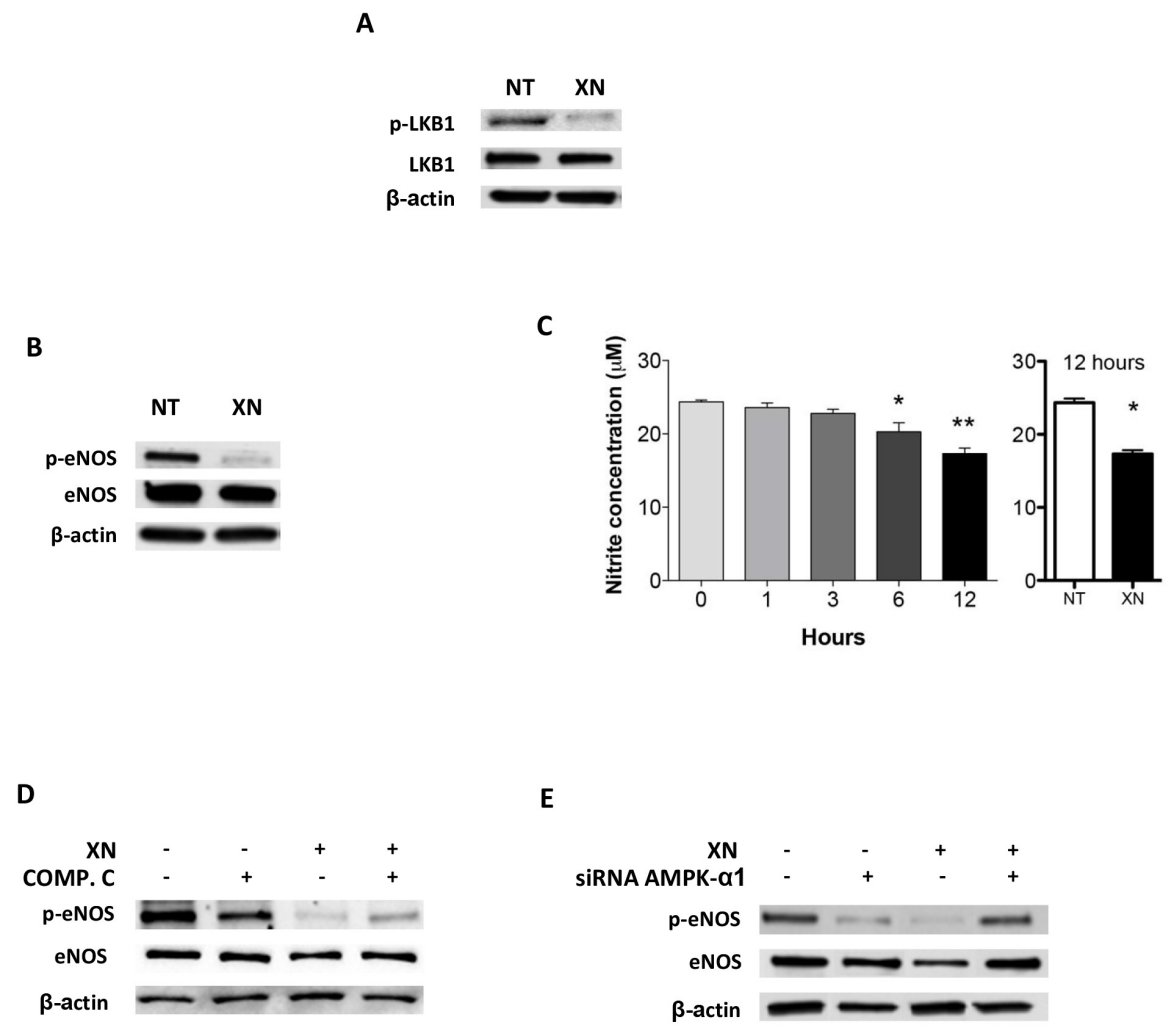
Dissection of upstream and downstram AMPK signals upon upon XN administration to human endothelial cells **A.** HUVEC were treated with 10 μM XN for 1 hour and LKB1 and phospho-LKB1 (Ser428) levels were analyzed by Western blotting. **B.** HUVEC were treated with 10 μM XN for 1 hour. Total and phospho-eNOS (Ser1177) were analysed by western blotting; **C.** Nitrite concentration following XN treatment was measured in HUVEC treated with 10 μM XN at different times up to 12 hours. Data are expressed as the mean± standard deviation of values from two independent experiments (*p<0,01; Student's t-test); **D-E.** HUVEC were pre-treated with 20 μM AMPK-inhibitor Compound C for 30 min **(D)** or transfected with AMPKα1-specific siRNA for 48 hours **(E)** then treated with 10 μM XN for 1 hour. Total and phospho-eNOS (Ser1177) were analyzed by western blotting. Anti- β-actin antibody was used as loading control.

**Figure 5:**
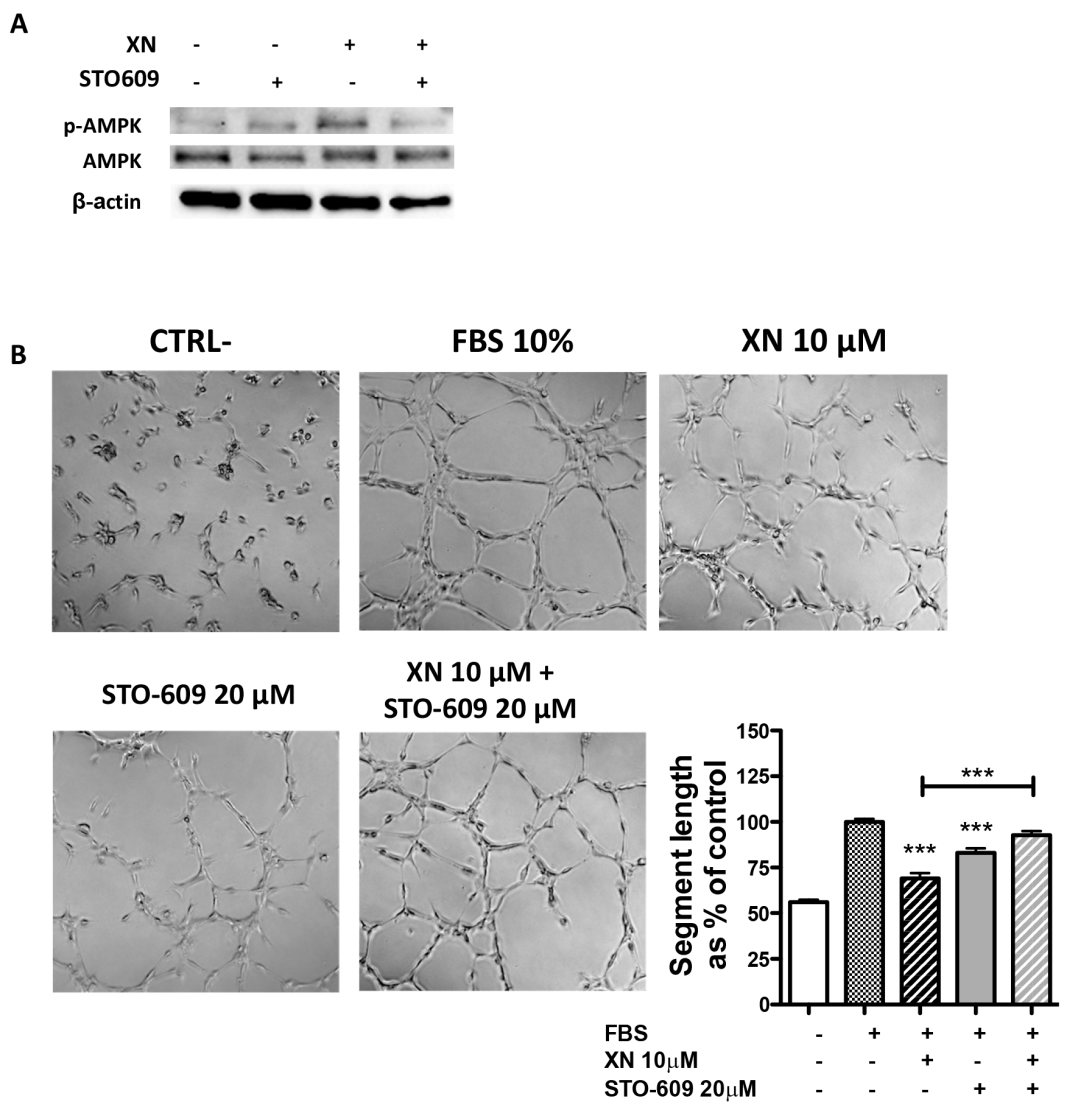
The role of CaMKKβ in XN-induced AMPK activation **A.** Cells were pre-treated with the CaMKKβ inhibitor, STO-609 (20μM; 30 min), followed by treatment with 10 μM XN. Phospho-AMPK and total AMPK protein levels were analyzed by western blotting. Anti- β-actin antibody was used as loading control. **B.** HUVE cells were pre treated with STO-609 (20 μM) for 30 and then treated with 10 μM XN. Microphotographs were taken at 10X magnification, representative images are shown. Capillary-like tube length was quantified by Angiogenesis analyzer ImageJ tool kit. Data are expressed as mean±SEM of the percentage of control values from independent experiments. Statistical significance (****p<0.0001; ***p<0.001; **p<0.01; *p<0.05; One-Way ANOVA) are with respect to control or as indicated by the bars.

AMPK is directly activated mostly by two mechanisms: decreased ATP/AMP ratio, leading to activation of LKB1, or Ca^2+^ dependent activation of CaMKKβ [30, 32]. To elucidate XN-induced molecular mechanisms upstream of AMPK activation, we assessed the contribution of these two molecular mediators in endothelial cells, following treatment with XN. Although XN significantly reduced intra-cellular ATP levels in HUVEC (Supplementary Figure S1F), it did not increase, and actually reduced, the levels of LKB1-Ser428 phosphorylation, without affecting LKB1 expression levels (Figure 4A). These data suggest that LKB1 is not able to activate AMPK upon treatment of endothelial cells with XN. We therefore investigated, as alternative pathway, the involvement of CaMKKβ in XN-induced AMPK activation. We treated HUVEC with STO-609, a selective CaMKKβ inhibitor, in presence or absence of XN or vehicle. Treatment with STO-609 (20 μM) for 30 minutes prevented AMPK phosphorylation at Thr172 induced by XN (Figure 5A). In line with these data, CaMKKβ inhibition by STO-609 prevented the reduction of tube formation following XN treatment (Figure 5B) and reverted XN inhibition of HUVE cell migration (Supplemental Figure S2). These data indicate that XN activates AMPK through CaMKKβ, which mediates XN-induced anti-angiogenic activities.

### XN inhibits eNOS activation in an AMPK dependent manner

Activation of AMPK may result in phosphorylation and induction of eNOS [38]. Since eNOS is both a target of AMPK and a modulator of angiogenesis, we evaluated the involvement of eNOS downstream of AMPK activation by XN in endothelial cells. Administration of XN for 1h led to a reduction of eNOS phosphorylation at Ser1177 (Figure 4B). Since nitrite and nitrate anions are the final products of Nitric-oxide (NO) oxidation and their presence in the media reflects the endogenous eNOS activity, we quantified the concentration of nitrite by the Griess assay following XN administration. Treatment of HUVEC with XN decreased NO levels at 12h as compared to untreated control, whereas NO levels were quite stable in controls in 12h time frame (Figure 4C). This is consistent with the necessary time occurring between protein activation by phosphorylation and the accumulation of detectable levels of the final products.

To verify whether AMPK is involved in XN-induced reduction of eNOS activity, we analyzed eNOS phosphorylation by western blotting in HUVEC in presence of XN and Compound C, an inhibitor of AMPK (Figure 4D), and by down-regulating AMPK-α1 expression with an AMPK-α1-specific siRNA (Figure 4E). Treatment with 20 μM Compound C inhibited basal eNOS phosphorylation and partially reverted the reduction of phospho-eNOS by XN (Figure 4D). Likewise, siRNA knock-down of AMPK-α1 abolished the decrease phospho-eNOS upon XN treatment (Figure 4E). Overall these data indicate that XN induces a repression of eNOS activity and that AMPK mediates this mechanism.

### XN independently modulates AMPK and AKT activities

We previously demonstrated that XN inhibits TNF-α-induced AKT phosphorylation in human endothelial cells [19]. AKT is a serine/threonine kinase supporting endothelial cell migration, with the ability to directly activate eNOS in an AMPK-dependent manner. AMPK and AKT are also responsible for the activation of the mTORC1 complex. AKT is activated by phosphorylation of Ser473 and Thr308. We therefore investigated the involvement of AKT pathways in the anti-angiogenic activity exerted by XN and we investigated the potential cross-talk with AMPK.

**Figure 6:**
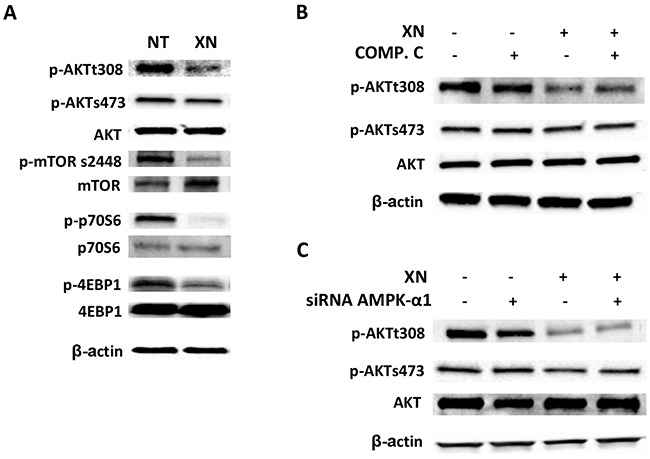
XN independently modulates AMPK and AKT activities **A.** HUVEC were treated with 10 μM XN for 1 h and the phosphorylation of AKT and its downstream targets mTOR (Ser2448), p70S6k (Thr389), 4EBP1 (Thr37/46) was assessed by Western Blotting; **B-C.** HUVEC were either pre-treated with 20 μM AMPK-inhibitor Compound C for 30 min (B) or transfected with AMPKα1-specific siRNA for 48h **(C)** then treated with 10 μM XN. AKT phosphorylation at Ser308 and Ser473 was analyzed by western blotting. Anti- β-actin antibody was used as loading control.

Treatment of HUVEC with 10μM XN for 1h induced a substantial decrease in Thr308-AKT phosphorylation, but failed to alter phospho-Ser473 levels (Figure 6A). To confirm the effects of XN on AKT pathway, we evaluated the phosphorylation of mTOR at Ser2448 and activation of its down-stream target, p70S6K and 4EBP1. XN treatment decreased the phosphorylation of mTOR, p70S6K and 4EBP1, in accordance with the observed decrease in AKT phosphorylation at the same time points (Figure 6A).

To evaluate a potential role of AMPK in XN-induced decrease of phospho-AKT levels, we inhibited AMPK activity using Compound C or AMPK-α1 siRNA in HUVE cells in presence or absence of XN. Direct inhibition of AMPK by compound C or siRNA knock-down of AMPK-α1 did not influence AKT phosphorylation after XN treatment (Figure 6B and 6C). Taken together, these data suggest that XN inhibits the AKT/mTOR pathway independently of its action on AMPK.

## DISCUSSION

Anti-angiogenic activities of several natural products found in vegetables, plants, spices, herbs and fruits such as carotenoids, polyphenols and terpenoids, and their synthetic counterparts have been observed. These compounds are of particular interest in clinical applications as they are easily available, often included in every day-diets and display low toxicity. Many of these compounds exert anti-oxidant [41], anti-proliferative, and pro-apoptotic effects on a variety of cancer cells, including leukemia, prostate, breast, colon, brain, melanoma, and pancreas.

Flavonoids are the most abundant polyphenols in the diet and several reports have shown the anti-tumor activity is exerted both on tumor cells and on the surrounding microenvironment in particular demonstrating anti-angiogenic, angiopreventive activities. Xanthohumol is among the chemo-preventive natural compounds with the ability to target the tumor vasculature. *In vitro*, we and others have shown that XN negatively interferes with endothelial cell growth, migration, invasion and the ability to form a network of tubular-like structures [19]. *In vivo*, XN reduces the number of vessels in matrigel plugs and wound healing assays [17, 42] and inhibits tumor-associated angiogenesis in different contexts [16, 18]. In this work we confirm the ability of XN to inhibit human endothelial cell functions and we show that XN anti-angiogenic related activities are stronger than those exerted by EGCG, a well-characterized anti-angiogenic compound found in green tea. We demonstrate that AMPK is a target of XN and a crucial mediator of the anti-angiogenic effects in human endothelial cells. We also elucidate the molecular mechanisms, identifying both intermediate and downstream players.

First, we confirmed the ability of XN to decrease endothelial cell functions *in vitro*. We used different XN dosages and time points and focused on 10μM, a dose sufficient to decrease endothelial cell migration, invasion and tube formation without significantly reducing cell proliferation or viability. This is consistent with other *in vitro* and *in vivo* studies showing a strong reduction of cell viability with 20μM XN [19, 20]. At the same doses, XN is more effective than EGCG, which is a known potent preventive agent for tumor invasion and angiogenesis [43]. Part of the anti-angiogenic activity of XN is exerted through inhibition of NF-κB activation and IκBα phosphorylation, and subsequent blockage of NF-κB translocation to the nucleus [18]. In addition, XN inhibits phosphorylation of endothelial AKT [19]. The AMPK pathway has emerged as another molecular pathway playing a major role in angiogenesis. Our data show that AMPK is required for angiogenic activities in basal conditions in line with the current literature [37, 44]. However, AMPK activation is associated with the inhibition of angiogenesis induced by natural product-derived compounds used in clinical settings, such as metformin [36, 45, 46], salicylate (the active form of aspirin) [47], EGCG [24], quercetin [25] and resveratrol [26]. Our data show that XN activates AMPK and that AMPK activation is necessary for the anti-angiogenic effects induced by XN, as the down-regulation of AMPK reverts endothelial cell ability to migrate and invade upon treatment with XN. To the best of our knowledge, this is the first report demonstrating that AMPK is a functional target of XN in human endothelial cells. This is in line with recent observations showing that XN activates AMPK in mouse liver cells and mouse embryonic fibroblasts [48, 49]. We also investigated the mechanisms through which XN leads to AMPK activation. Genetic and biochemical evidence supports the idea that LKB1 is the major AMPK-kinase regulator in several mammalian cell types in response to changes in the AMP/ATP ratio [33, 34, 50]. Pharmacological drugs decreasing ATP levels lead to a reduction in the ATP/AMP ratio, which finally activates AMPK [28, 51]. Since XN interferes with ATP production [21] and ATP is required for tubulogenesis processes at early time points [52], it has the potential to activate AMPK through LKB1 in endothelial cells. However, we found that XN decreases LKB1 phosphorylation instead of inducing it, excluding a role for LKB1 in AMPK activation by XN. The imbalance of AMP:ATP and ADP/ATP ratio can also activate other kinases, such as CaMKKβ [53], a well known regulator of AMPK. Our data support the involvement of CaMKKβ as the main kinase responsible for AMPK activation in endothelial cells following treatment with XN. This is in line with the ability of XN to increase intra-cellular calcium observed in other systems [54, 55]. Moreover, CaMKKβ inhibition by STO-609 partially impaired XN mediated anti-angiogenic activity on HUVE cells *in vitro*. AMPK activation in endothelial cells leads to the phosphorylation of endothelial nitric oxide synthase (eNOS) [38, 56]. We observed a reduction of activated eNOS in XN treated cells, accompanied by a decrease in the release of NO. Other reports show the ability of XN to inhibit NO production by decreasing iNOS (inducible-NOS) activity or expression [57]. *In vivo*, XN ingestion in rats decreases NO release (77.06%) [42]. Our data are consistent with these findings.

**Figure 7:**
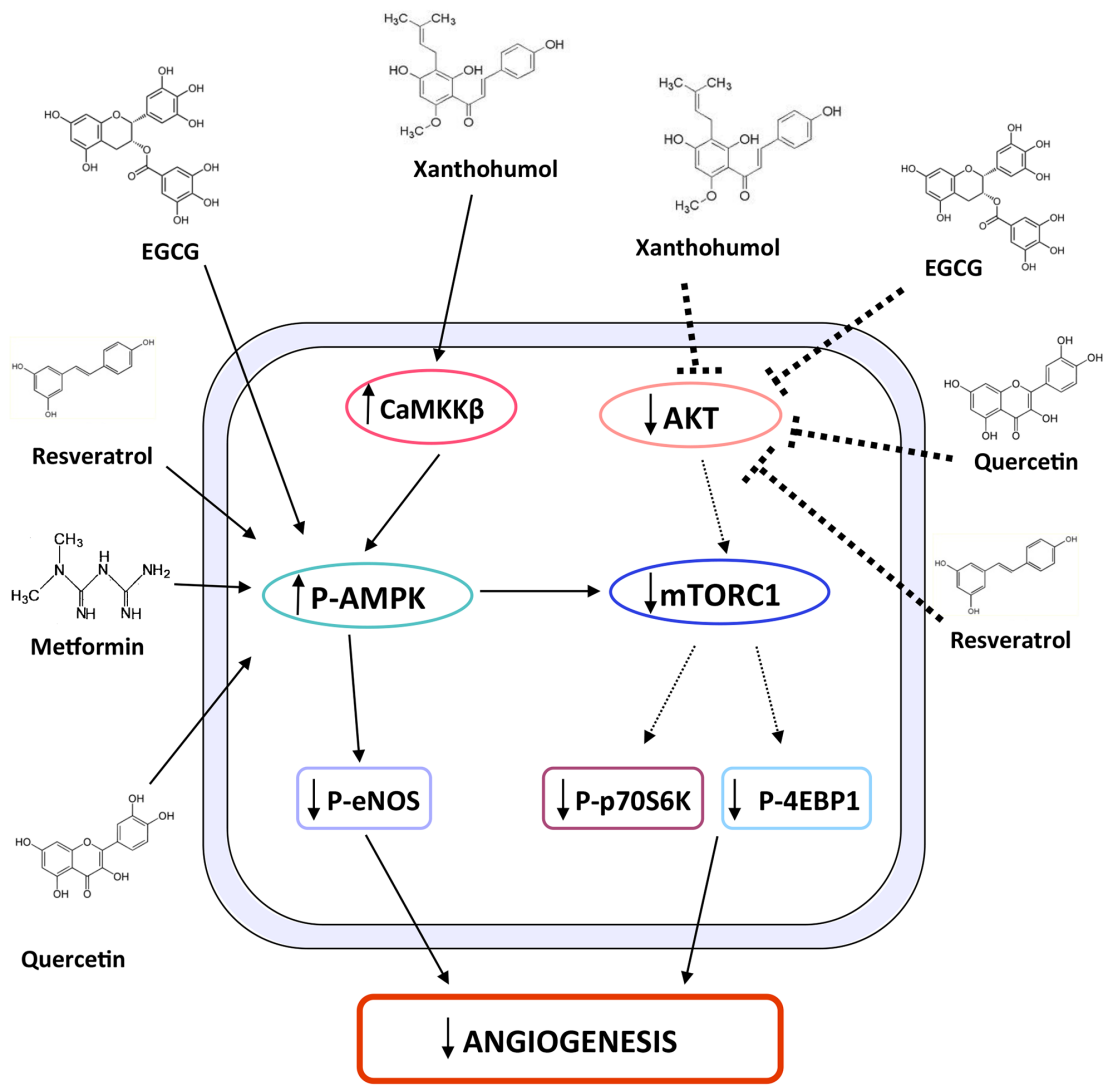
Schematic representation of XN-induced molecular pathways leading to anti-angiogenic effects The schematic diagram shows the molecular pathways by which XN exerts anti-angiogenic activities. In endothelial cells XN treatment activates AMPK through CaMKKβ, which in turn inhibits eNOS activation. In parallel, XN inhibits AKT phosphorylation and consequently the downstream pathway constituted by mTORC1, p70S6K and 4EBP1. The two mechanisms lead to inhibition of angiogenesis by XN as well as other flavonoids, including EGCG, resveratrol and quercetin, which have been shown to act through similar mechanisms.

Extensive data suggest the existence of a cross-talk between AMPK and AKT signaling in angiogenesis [8]. A mutual suppression of both AKT and AMPK pathways by chemo-preventive agents, such as quercetin, has been reported [58]. Resveratrol suppresses PI3K signaling by binding to the ATP-binding site of PI3K [59]. In a previous work, we have shown that XN inhibits AKT activation in endothelial cells, possibly through the inhibition of the PI3K pathway [18]. AKT is activated by phosphorylation of Ser473 and Thr308, by mTORC2 and PI3K, respectively. Here we observed that XN decreases AKT phosphorylation at Thr308. Following treatment with XN, we did not observe any modulation of Ser473 phosphorylation on AKT [60]. Consistent with XN-induced inhibition of PI3K/AKT/mTORC1 axis, we found inhibition of mTOR pathway in endothelial cells treated with XN. Our data suggest that XN mechanism of action in endothelial cells involves two distinct molecular cascades. On one hand, XN activates CaMMK/AMPK/eNOS axis. On the other hand, XN inhibits AKT/mTORC1 axis. These two mechanisms of action seem to proceed in parallel, leading to inhibition of angiogenesis (Figure 7). The doses of XN employed in our study are in line with those used in our previous publications and in clinical trials (https://clinicaltrials.gov/ct2/show/NCT01367431?term=xanthohumol&rank=2; https://clinicaltrials.gov/ct2/show/NCT02432651?term=xanthohumol&rank=1).

Overall, our study elucidates the molecular mechanisms of the anti-angiogenic activity of XN. Given the ability of tumors to resist anti-angiogenic therapies by activating alternative pathways, there is an increasing need to therapeutically block tumor angiogenesis by targeting multiple anti-angiogenic pathways. Polyphenols, such as EGCG, affect the activity of multiple pathways and mediators involved in the carcinogenesis process and were previously suggested as valuable inhibitors of cancer cell growth and angiogenesis [61]. Our results provide evidence that XN might be a more potent and suitable compound for the inhibition of AMPK and AKT mediated inhibition of angiogenesis both in therapeutic and prevention settings. Our results also suggest a potential commercial interest of XN as dietary supplement in a chemopreventive/angiopreventive approach.

## MATERIALS AND METHODS

### Reagents

Xanthohumol ((2E)-1-[2,4-dihydroxy-6-methoxy-3-(3-methyl-2-buten-1-yl)phenyl]-3-(4-hydroxyphenyl)-2-propen-1-one) was purchased from Alexis Biochemicals (San Diego, CA, USA). Epigallocatechin-3-gallate (EGCG) was purchased by Sigma Aldrich (Sigma-Aldrich, St Louis, MO, USA). STO-609 was purchased from Sigma-Aldrich (St Louis, MO, USA) and Compound C from CalbioChem (Darmstadt, Germany). The ON-TARGET plus SMART pool siRNAS targeting AMPK α1 (PRKAA1 L-0502700) and ON-TARGET plus Nontargeting Pool negative control siRNA (D-001810) were purchased from Dharmacon (Lafayette, CO). Lipofectamine RNAiMAX (catalog number 13778-075) was purchased from Invitrogen (Eugene, OR).

### Cell lines and cell culture medium

Human umbilical vein endothelial cells (HUVEC) were obtained from PromoCell (Heidelberg, Germany) and cultured from passage 4 to 6 at 37 °C in 5 % CO_2_ in M199 medium (Sigma Aldrich) supplemented with 10% heat-inactivated fetal bovine serum, 1% glutamine, fibroblast growth factors (1μg acidic-fibroblast growth factor plus 1μg basic-fibroblast growth factor/100 ml, PeproTech London UK), epidermal growth factor (1μg/100 ml, PeproTech), heparin (10 mg/100 ml, Sigma Aldrich) and hydrocortisone (0.1 mg/100 ml, Sigma Aldrich). HUVEC were seeded on 0.1% gelatin coated flasks.

### MTT assay

*In vitro* cell proliferation/viability was measured by the MTT test at different time points. 1000 cells/well were plated into 96-multiwell plates in complete medium. Following adhesion, medium was replaced with fresh medium containing the different treatments or vehicle (DMSO in medium). XN and EGCG were used in a concentration range from 2.5 to 40 μM, up to 96 hours. 3 hours before each time point, MTT reagent (3-(4,5-dimethylthiazol-2-yl)-2,5-diphenyltetrazolium bromide; Sigma Aldrich, Milano) was added to the wells and plates were incubated at 37°C. At the indicated time points, absorbance at 540 nm was then measured by a FLUOstar spectrophotometer (FLUOstar Omega BMG LABTECH).

### Apoptosis assay

Apoptosis was evaluated by 7-AAD staining, followed by flow cytometry analysis. HUVE cells were plated at a density of 2.5 × 10^5^ cells/well on 6-well plates and grown overnight. The following day, the cells were treated either with XN or EGCG in a concentration range from 5 to 20 μM, up to 96 hours. DMSO in medium or medium alone were used as vehicle. At each time point, cells were harvested, counted, transferred into flow tubes, pelleted, resuspended in 50 μl of PBS and stained with the 7-aminoactinomycin-D (7-AAD) dye. Samples were analyzed by flow cytometry within 1 hour using a FACSCantoII (BD). The proportion of viable (7-AAD-negative) cells was analyzed using the BD FACSDiva Software 6.0 and expressed as a percentage of the total cell number, excluding debris.

### Cell cycle assay

Assessment of cell cycle was evaluated with Propidium Iodide (PI) staining followed by flow cytometry. 2 × 10^5^ HUVE cells were plated on 6-well plates. The following day cells were treated either with xanthohumol or EGCG in a concentration range from 5 to 20 μM, for 24 or 48 hours. DMSO in medium or medium alone were used as vehicle controls. Cells were fixed and permeabilized with 70% ice-cold ethanol for 1 hour at −20°C, then washed twice with cold Phosphate Buffer Solution 1X and stained for 40 minutes at 37°C with PI solution (50 μg/ml PI in H_2_O, 0.1% Triton-X100, 0.1% trisodium citrate dehydrate, 6.25 μg/ml RNase A). Cells were analyzed by flow cytometry within 1 hour using a FACSCanto machine (BD) and analyzed with BD FACSDiva Software 6.0.

### Migration and invasion assays

Chemoinvasion and chemotaxis assay was performed as previously described [62, 63]. Briefly, polycarbonate membrane filters with 8 μm pore-diameter (Whatman, GE Healthcare Europe GmbH, Milano) were pre-coated with collagen IV (50 μg/mL, Sigma Aldrich Milano) for chemotaxis assay or matrigel (1 mg/mL, BD Biosciences, Milano) for chemoinvasion assay and placed in modified Boyden chambers (Neuro Probe, Gaithersburg, MD, USA). HUVECs (5×10^4^), pre-treated with XN or EGCG (range 5 to 20 μM) for 24h, were washed with PBS, resuspended in serum-free medium and placed in the upper compartment. Serum (10% in M199) was used as chemoattractant and added to the lower compartment of the chambers. Cells were incubated for 6h (chemotaxis) or 24h (chemoinvasion) at 37°C, filters were then recovered, cells on the upper surface were mechanically removed with a cotton swab. For the experiments using the STO609 compound (20 μM), cells were pre treated for 30 minutes, then treated with 10 μM XN for 1 hour. Migrating or invading cells were fixed with absolute ethanol and stained with DAPI (Vectashield, Vector Laboratories, Orton Southgate, Peterborough, United Kingdom). Cells were counted in a double-blinded manner in five consecutive fields with a fluorescent microscope (Nikon Eclipse Ni). All experiments were performed three times in duplicate.

### Matrigel morphogenesis assay

When cultured on a tree-dimentional membrane-basement matrix, HUVECs are able to form capillary like structures, mimicking the events occurring during vessel lumen formation *in vivo*. A 24-microwell plate, pre-chilled at −20°C, was carefully filled with 300 μl/well of liquid matrigel (10 mg/ml, BD Biosciences, Milano) at 4°C with a pre-chilled pipette. The matrigel was then polymerized for 1 h at 37°C. 5 × 10^4^ HUVEC, pre-treated with XN or EGCG (range 5 to 20 μM) for 24h, were suspended in 1 ml of complete medium and layered on the top of the polymerized matrigel. For the experiments using the STO-609 compound (20 μM), cells were pre-treated for 30 minutes, then treated with 10 μM XN for 1 hour. After 6 h of incubation at 37°C, the formation of capillary-like networks was examined under an inverted microscope (Nikon Eclipse TS100), equipped with charge-coupled device optics and a digital analysis system. The length of segments were quantified by the ImageJ software, using the “Angiogenesis Analyzer” tool [64].

### Transfection of HUVEC with AMPK α1 specific siRNA

Transient transfection of siRNA was performed using Lipofectamine RNAiMAX, according to the manufacturer's protocol. Briefly, on day 1, HUVEC were seeded at a density of 2 × 10^5^ per well in a gelatin pre-coated six-well plate, without antibiotics. The following day, cells were transfected with 10 nM siRNA for 6 h. Transfection efficiency was verified 24 and 48 h later by western blotting.

### Western blotting

HUVEC were grown in complete medium and treated with increasing concentrations of XN (1–20 μM). Cells were then washed with PBS 1X at and snap-frozen at −80°C. After thawing, cells were placed in lysing buffer (Cell Signaling Technology, Beverly, MA) and total lysate was obtained. Protein concentration was determined by the Bradford Protein Assay (Bio-Rad, Hercules, CA). Equal amounts of proteins for each sample were resolved on 4-20% sodium dodecyl sulfate–polyacrylamide gel electrophoresis and blotted onto polyvinylidene fluoride membranes (Amersham Biosciences, Otelfingen, CH). Following blocking with 5% non-fat milk powder (wt/vol) in Tris-buffered saline (10mM Tris–HCl, pH 7.5, 100mM NaCl, 0.1% Tween-20) for 1h at room temperature, membranes were incubated with primary antibodies directed against the following human antigens: β-actin (Sigma Aldrich), AMPKα1, phospho-AMPKα (Thr172), total and phospho-ACC, total and phospho-eNOS, total and phospho-LKB1, total and phospho-mTOR(Ser2448), total and phospho-AKT (Thr308 and Ser473), total and phospho-p70S6k, total and phospho-4EBP1 (all purchased from Cell Signaling Technology, Danvers, MA). The antibodies were diluted in 2% bovine serum albumin–Tris-buffered saline–0.1% Tween according to the manufacturer's instructions. The bound antibodies were visualized by horseradish-peroxidase-conjugated secondary antibodies and an enhanced chemiluminescence detection system from Amersham Biosciences (Pittsburg, PA).

### Griess assay

Nitric oxide (NO) release was indirectly analyzed by measuring nitrite (NO_2_^−^), a stable and non-volatile breakdown product of NO. HUVE cells were grown in complete medium and treated with 10 μM XN in a time range from 1h up to 12h. At the indicated time points, the medium was collected. The assay was performed according to the manufacturer's protocol for Griess Reagent System (Promega, Milan, Italy). Briefly, 50 μl of each sample was transferred to a 96 multiwell plate. Each sample was then incubated with 50 μl of Sulfanilamide Solution for 10 minutes, followed by a second incubation with 50 μl of N-1-napthylethylenediamine dihydrochloride (NED) Solution for 10 minutes. Absorbance was measured at 540 nm by using a FLUOstar spectrofotometer (FLUOstar Omega BMG LABTECH) and then compared using a Nitrite Standard reference curve.

### ATP assay

The CellTiter-Glo Luminescent Cell Viability Assay (Promega, Milan, Italy) was used to measure the amount of ATP produced by endothelial cells. HUVEC (6000/well) were plated into 96-multiwell plates in complete medium. Cells were then serum-starved for 18 hours. Then, XN was added to each well to a final concentration of 10 μM. Control cells (NT) were treated with vehicle alone (DMSO). 1 h after treatment, 100 μL of CellTiter-Glo Reagent, previously prepared by mixing the CellTiter-Glo Buffer and the lyophilized CellTiter-Glo Substrate, was added to each well. Luminescence was read by using NanoLuc Luciferase Ready (GloMax Discover, Promega, Milan, Italy).

### Statistical analysis

Data are expressed as means ± SEM. The statistical significance between multiple data sets was determined by one-way ANOVA using Graph-Pad PRISM FACS data were analyzed by the FACSDiva6 software. ImageJ software was used for western blotting quantification.

## SUPPLEMENTARY FIGURES


