# Supplementary material for: Hop derived flavonoid xanthohumol inhibits endothelial cell functions via AMPK activation
Source: Oncotarget. 2016 Aug 1;7(37):59917–31. doi: 10.18632/oncotarget.10990 (PMC5312358; doi:10.18632/oncotarget.10990)
Supplement: Supplementary file 1 [file oncotarget-07-59917-s001.pdf]

# Hop derived flavonoid Xanthohumol inhibits endothelial cell functions via AMPK activation

## SUPPLEMENTARY FIGURES

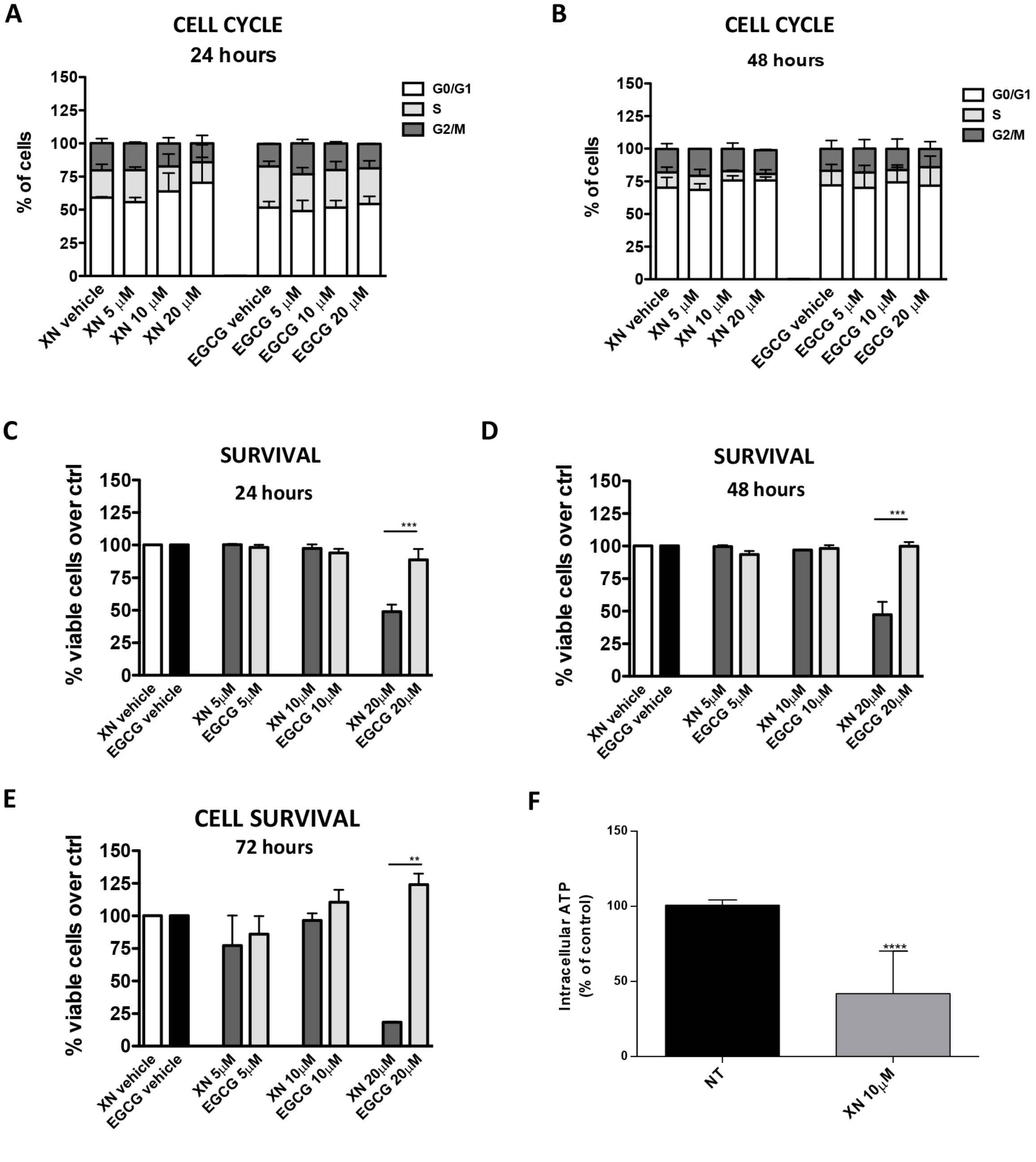


**Supplementary Figure S1: Flow cytometry assessment of cell cycle and cell survival in HUVEC upon treatment with XN or EGCG and quantification of intra-cellular ATP upon treatment of HUVEC with XN.** HUVEC were treated with XN and EGCG at different doses (5-10-20 µM) or vehicle. **A–B.** Cell cycle was evaluated 24h and 48h following treatments by flow cytometry analysis after propidium iodide staining. Histograms show the distribution of cell populations in each phase of the cell cycle. **C–E.** Cell survival was evaluated 24h, 48h and 72h following treatments by flow cytometry analysis of 7AAD staining. 7ADD-negative (viable) cells are reported as % of untreated control. **F.** ATP levels were measured in HUVEC treated with 10 µM XN for 1h. Data are expressed as the mean ± standard deviation from two independent experiments in triplicate (***p<0.001; **p<0.01; *p<0.05; Student's t-test; ns: not significant).

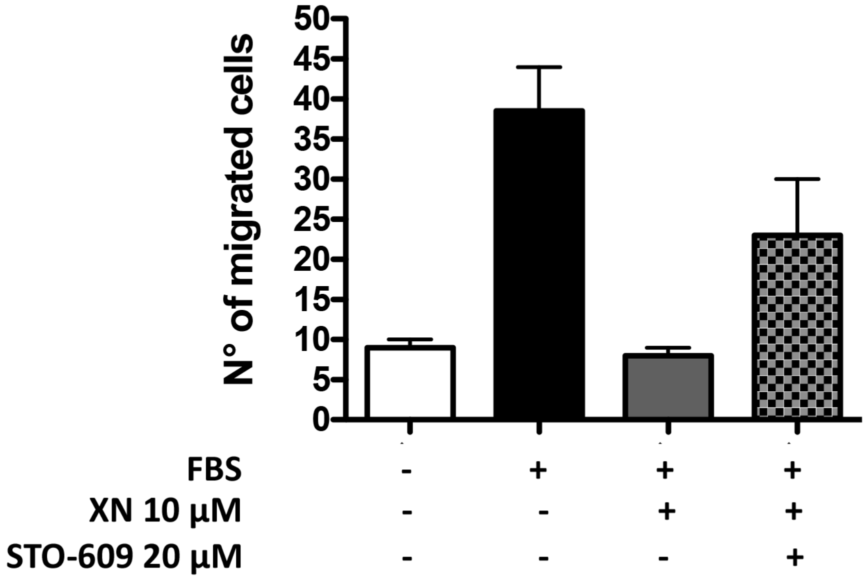


**Supplementary Figure S2: The role of CaMKKβ inhibition in XN-induced altered endothelial cell migration.** HUVEC were pre-treated with the STO609 (20 µM) for 30 minutes, followed by exposition to XN (10 µM) for 1 hour, and then seeded in serum free medium in the upper compartment of Boyden chamber. Cell migration was measured at 6h.
